# Natural Products from Madagascar, Socio-Cultural Usage, and Potential Applications in Advanced Biomedicine: A Concise Review

**DOI:** 10.3390/molecules26154507

**Published:** 2021-07-27

**Authors:** Camillo La Mesa, Oliarinony Ranalison, Lovasoa N. Randriantseheno, Gianfranco Risuleo

**Affiliations:** 1 Department of Chemistry “Stanislao Cannizzaro”—University of Rome La Sapienza, Piazzale Aldo Moro 5, 00185 Rome, Italy; camillo.lamesa@uniroma1.it; 2 Animal Biodiversity and Zoology, Faculty of Sciences, University of Antananarivo, BP 906, Antananarivo 101, Madagascar; ranalison.oliarinony@gmail.com; 3 Department of Fundamental and Applied Biochemistry, University of Antananarivo, BP 906, Antananarivo 101, Madagascar; lovasoanomena@pasteur.mg; 4 Department of Biology and Biotechnologies “Charles Darwin”—University of Rome La Sapienza, Piazzale Aldo Moro 5, 00185 Rome, Italy

**Keywords:** natural products, Madagascan active principles, nano-delivery

## Abstract

Natural products endowed of biological activity represent a primary source of commodities ranging from nutrition to therapeutic agents, as well as cosmetic tools and recreational principles. These natural means have been used by mankind for centuries, if not millennia. They are commonly used all over the world in socio-economical contexts, but are particularly attractive in disadvantaged areas or economically emerging situations all over the world. This is very likely due to the relatively easy recovery of these bioactive principles from the environment, at a low if any cost, as well as ease of administration and the general popular compliance concerning their consumption/ingestion. In this concise review, we focus on some popular bioactive principles of botanical origin which find a wide use in the Madagascan populations. However, due to space limitations, only some of the most common and largely diffused principles in this country are considered. Finally, a possible nanotechnological administration is discussed in the case where a potential therapeutic usage is envisaged.

## 1. Introduction

Since the dawn of the ages, mankind has catered from nature means of survival and support: food, textiles and furs for shelter and wood for housing (though in more recent eras), or for cooking fire as well as for defense from cold and adverse climates. Certainly, nutrition was based on hunting meat, but also on vegetable commodities very early in human civilization, and their availability played an important role in daily life. One can imagine that at the very beginning, herbs and fruits were harvested in a bulk fashion no matter whether palatable or not: certainly, the habit of eating what was found in nature had to follow a trial-and-error process. Obviously, the food scavenger learned at his/her own expense that the mushroom *Amanita phalloides* or the castor bean of *Ricinus communis*, just to mention two highly lethal plants, where to be avoided as food. In fact, they quite efficiently, rapidly, and painfully terminated the life of the unfortunate, though unaware, consumer. It is almost impossible to establish when humans started to use natural products for purposes different from nutrition, such as recreation or cosmetics. To remain among mushrooms and succulents, somebody must have realized at some point that the *Amanita muscaria* (fly amanita) or the *Lophophora williamsii* (peyote) had powerful hallucinogenic and psychoactive properties; they could therefore be used for socializing, or to take psycho-trips and, last but not least, as aphrodisiac help tools [1,2,3].

Natural plant and animal products (as well as their selection) establishes the end of the hunter-gatherer lifestyle and coincides with the start of routines of agricultural practices and animal husbandry. Consequently, humans became bound to a stable site. This, in a way, permitted the sedentary man or woman to collect and use fruits or roots available on site. Breeding started too, with the sedentary habit and domestication of the first agricultural plants, dated 11,000 to 9000 years ago [4,5,6].

In any case, bioactive natural products are widely diffused in the so-called alternative or popular medicine practice; possibly, one of the best-known examples is represented by the neem tree and its products. The usage of neem fruits, leaves and even bark is described in religious Hindu works and in the Ayurvedic medicine, where it is indicated by the Sanskrit name of “sarva roga nivarini” (universal healer of all illnesses). Allegedly, the Mahatma Gandhi used to meditate and pray under the foliage of a neem tree to find inspiration and tranquility and, actually, consumed neem leaves with his daily diet [7], see also the web site http://www.natureneem.com/index.htm (accessed on 23 June 2021).

However, in the light of the updated scientific literature, the all-healing, all-purpose character of neem products remains controversial [8]. Apart from any consideration, the usage of natural products ranges from nutraceutics to human/veterinary health care and therapies as well as to cosmetics. Just to give an idea about the number and diffusion of natural substances one should be aware of, a search in a common literature databank, using the keyword “natural products”, will score about 68,000 entries (as of June 2021). Therefore, this topic is of utmost interest for the general reader, but also for related economical and industrial enterprises.

The products in question are defined as natural, since they are found in nature and usually do not require any modification or intervention by human activity, if not to render them administrable to the interested user; usually crushing, extracting by diverse agents or physical treatment (such as drying and/or fermenting) are the only adopted manipulations. In modern times, thanks to the development of biomolecular technologies, pharmaceutical methods of analysis and screening, these products have become the source of new therapeutic means to treat several pathologies. In the past, great attention was dedicated to the identification of natural substances to alleviate and improve the human condition; nowadays chemistry works essentially on “prototype structures” that are at the basis of synthetic drugs developed from their natural “ancestor” molecules. One very important aspect that should be considered is the possibility of transferring bioactive substances by means of nano-vectors; this last aspect will be examined in the final part of this work. For a rather extensive illustration of the biomolecular characteristics and for an updated “catalogue” of bioactive natural substances, the reader is pointed towards recently published works [9,10].

As mentioned above, natural products find a use in many fields of daily life—this applies especially in areas of the world where traditional medicine is practiced for a number of different reasons; essentially, proximity and easy access to natural principles, fairly uncontaminated environment, and low-cost/benefit ratio of the product in question. It should be pointed out, however, that the use products of natural origin are also becoming ever more popular in areas of the economically advanced world. If this is due to a return to a healthier lifestyle or to a snob-like attitude is hard to tell. However, in any case, examining these socio-economic sides lies outside of the boundaries of this contribution.

According to the classification of Newman and Cragg [10], bioactive compounds can be divided into several different categories, for instance: 1. biological, usually large peptides or proteins either isolated from an organism/cell line or produced biotechnologically; 2. natural products, unmodified in structure, but semi- or totally synthetic; natural products or “botanical drugs”; 3. compounds derived from a natural product and semi-synthetic modification. The ones pertaining to this contribution fall essentially into categories 2 and 3. We would like to point out that only in the category of the so called “botanical drugs” or “nutraceutical substances” are a few thousands of compounds comprised.

In any case, Madagascar, among other emerging countries, is one of the best-known for the diffused usage of natural remedies of active principles of botanical origin: for sake of brevity, this concise review will focus on some of the most popular and widely used remedies of natural derivation in the Île Rouge, as it is also known in French. However, a beautiful and detailed compendium of Madagascan medicinal plants can be found in the book by Boiteau and Allorge-Boiteau [11]. This review focuses on some of the most commonly used bioactive principles of Madagascan origin, as to our knowledge; not many recent works and contributions about Malagasy bioactive products seem to have been newly published. Also, a potential nanotechnological delivery of these either pure or mixed products is envisaged, although the authors are aware that these advanced bio-medical strategies are, unfortunately, of difficult application in disadvantaged areas of our globe.

We would like to point out, furthermore, that as far as the Malagasy medicinal flora is concerned, we do not discuss here other very popular botanical remedies and foodstuff such as papaya, corossol (soursop) and lychee: this latter is possibly the most known and appreciated Madagascan fruit all over the world. This is not a negligence but is only for sake of brevity.

## 2. Some Common Madagascan Natural Products

### 2.1. Vonenina

**Figure 1:**
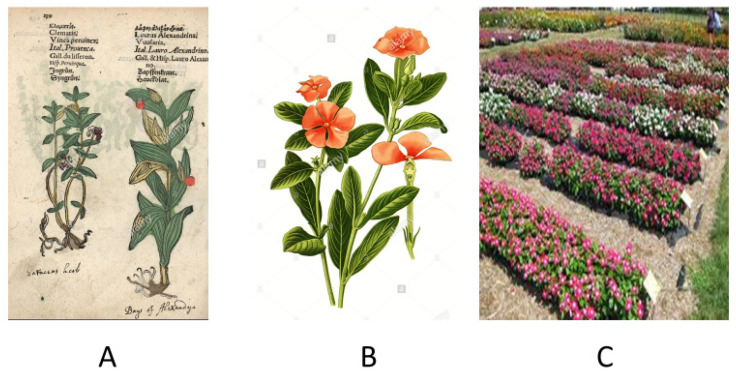
The plant of *Vinca rosea*. (**A**,**B**) Antique hand-drawn pictures of the plant *Vinca rosea*. These images are re-elaborated from Adam Lonicer’s Kräuterbuch or Herbal Handbook published in Frankfurt (Germany) in 1557. Adam Lonicer, also known as Adam Lonitzer or Adamus Lonicerus (1528–1586) was a German botanist, noted for his 1557 revised version of Eucharius Rösslin’s herbal (Adam Lonicer: Kräuter-Buch, 1703). However, despite the beautiful and suggestive character of these xylographic pictures, according to some historians and naturalists this work is to be considered as a forgery; for further details see: https://books.google.it/books?id=TDw_AAAAcAAJ&hl=de&pg=PP5&redir_esc=y#v=onepage&q&f=false (accessed on 23 June 2021). (**C**) Semi industrial plant nursery showing intensive cultivation of *Vinca rosea*.

**Figure 2:**
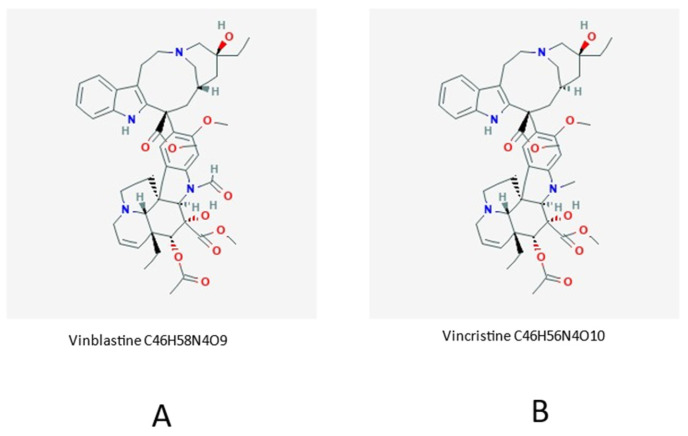
Chemical formulas of vinblastine (**A**) and vincristine (**B**).

Seven species of the ornamental plant periwinkle, out of the eight known ones, are endemic to Madagascar. Even though the plant is native to Madagascar, it has propagated to other geographical areas such as India, Sri Lanka, Sierra Leone, Malaysia, and Australia, to mention but a few. The plant has a number of different common names such as “bright eyes”, “graveyard plant”, “old maid” or “rose periwinkle maid”. The *Catharanthus roseus* (also known as *Vinca rosea*) is the source of an active principle known with the Malagasy name of *vonenina*. The plant has many other names in Madagascar, such as: *tsmatiririnina*, *befela* or *salotsa*, depending on tribal language and ethnic context such as Merina and Betsileo, which are the main, and historically speaking most influential, indigenous tribes of the Red Island. The Madagascan periwinkle has been known for a long time as a herbal medicine, although it has also found a very common usage as an ornamental plant. However, the active principles of this plant are not limited to the Madagascan popular pharmacopeia. As a matter of fact, Ayurvedic and Chinese traditional medicines recommend the extracts of roots and shoots as medicament for different diseases, among which are diabetes and malaria. However, if assumed orally, *C. roseus* can be extremely toxic to humans. In more recent times, vinca-alkaloids were purified from this plant, including vinblastine and vincristine. Presently, these natural compound derivatives have a very relevant role in human medicine. As a matter of fact, these molecules are used as chemotherapeutic agents in the treatment of different types of cancer, including leukemia and Hodgkin’s lymphoma. These molecules are used to treat including Hodgkin’s lymphoma, acute lymphocytic and myeloid leukemia, non-small cell lung cancer, bladder cancer, brain cancer, melanoma, as well as testicular cancer [12]. Administration is intravenous. Both drugs are included in the WHO list of Essential Medicines. http://apps.who.int/iris/handle/10665/325771 (accessed on 23 June 2021). World Health Organization model list of essential medicines: 21st list 2019. Geneva: World Health Organization). In any case, vinblastine and vincristine are derived from natural *Vinca* alkaloids, and are, chemically speaking, analogue molecules to those nowadays produced by chemical synthesis [13,14]. In tumor cells, vinblastine binds tubulin and thus inhibits microtubule assembly; this ends in cell cycle arrest in the M phase since disruption of microtubule assembly and proper formation of mitotic spindles as well as kinetochore are severely hindered. The separation of chromosomes during the mitotic anaphase is, consequently, dramatically impaired (See Figure 1 and Figure 2, for *Vinca rosea* pictorials and for the chemical generalities of the antitumor molecules derived from this plant). However, about 180 species of honeysuckle species exist, belonging to the genus Lonicera. They are diffused in North America and Eurasia. The term Lonicera was created by Karl von Linné (Karolus Linneus) in the mid-seventeenth century, adopting in Latin the name Lonitzer as tribute to the botanist Adam Lonitzer. These plants are also known under the common name of caprifolium, which derives from the Latin word goat (capra) and leaf (folium), referring to the habit that this ovine graze on the leaves of some of these plant species.

The close molecular/chemical analogy of the two active principles is evident. Vinblastine is commercially sold as Velban but is also known under different brand names. Vincristine (also named leurocristine) is found on the market under the brand name Oncovin but, as in the previous case, different brand names are commercially available.

### 2.2. Lantana and Marigold

Neither plant is actually native to Madagascar. However, bioactive products of both plants are applied in the Malagasy popular/traditional medicine. The native habitat of *Lantana camara* (common lantana) is Central, South America and American tropics; however, due to its extremely invasive character it has spread to over 60 countries worldwide, including temperate southern European areas. Other common names of *L. camara* are Spanish flag, big-sage (Malaysia), wild-sage, red-sage, white-sage (Caribbean), korsu wiri (korsoe wiwiri) (Suriname), tickberry (South Africa), West Indian lantana, umbelanterna, putus in Bengal and Gu Phool in the Assam region (India). This plant is known in Madagascar by different names, such as radriaka, falavelona or fankataviakoho, depending on the local languages. However, the definition of falavelona is also used for the plant *Jatropha curcas* (personal information received by one of the Authors, LNR, from diverse sources; therefore, one should be careful and avoid confusion). The plant was introduced from tropical America, possibly by voyagers and merchants, though the Northern coasts of the island have also been known for pirate activities which could have generated import and/or exchange of commodities. The wound healing properties of the juice of Madagascar’s *Lantana camara*, in any case, were discovered upon the vast and capillary spreading of this plant into the new habitat. The lantana leaf juice and essential oil constitute the basis for Radriaka, a commercial preparation produced in Madagascar. This is often confused with the drug Madécasol (or Madecassol, originally produced by Bayer), which is actually obtained from the *Centella* species. However, both preparations are utilized as a cicatrizant, in addition to other disparate discomfort situations such as antispasm, antiviral, emmenagogue, and febrifuge. Therefore, it is used to alleviate many conditions among which are: ulcers, wounds, cuts, chronic bronchitis and asthma, viral infections, as well as to induce and/or regulate menstruation. Also, an infusion (tea) is used as a beverage and wash to alleviate scabies and cure common colds. Tea and steam baths are used as an antipyretic and against shakes. See https://en.wikipedia.org/wiki/Lantana_camara (accessed on 23 June 2021) for further details.

The species *Tagetes minuta*, known with the common name marigold is an annual plant native to South America; it was introduced to Madagascar as an ornamental plant and is now naturalized throughout the highlands. As a matter of fact, it also grows at relatively high altitudes in the Antananarivo area and its surroundings such as Ankatso, Alasora and Ambohimangakely (the average altitude is about 1200 m above sea level). Oils of *Tagetes minuta* from various countries have been investigated for their chemical composition, showing large differences in percentages of the main compounds, and the composition of oils extracted from Madagascan marigold plant has been investigated in detail [15]. Essential oil preparations, in association with *Calendula officinalis* products, have been used in a number of podiatric conditions, including the hallux valgus or other skin lesions [15]. In particular, a decoction of leaves of *Tagetes erecta,* also commonly known as Aztec or African marigold, has traditionally been used for the treatment of malaria in Madagascar, while the roots are used for their insecticidal and nematocidal activities, possibly due the thienyl compounds found therein [16,17].

For more details about the complex taxonomy of marigold (*Tagetes* and *Calendula* genus) the reader is pointed towards: https://en.wikipedia.org/wiki/Tagetes, https://en.wikipedia.org/wiki/Calendula (accessed on 23 June 2021).

**Figure 3:**
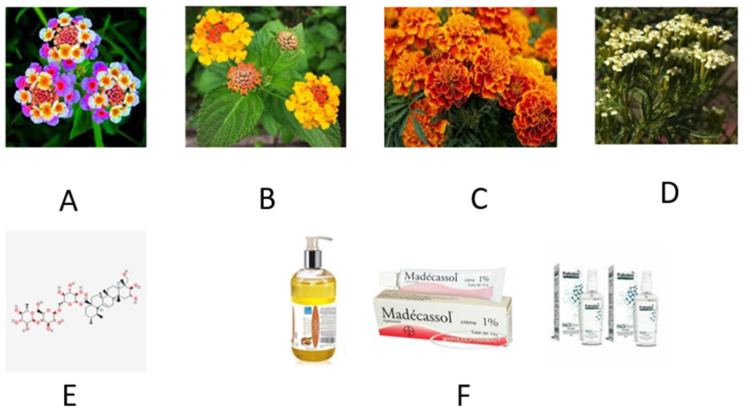
Generalities of the plant *Lantana Camara* and its products. (**A**,**B**) Inflorescences of *Lantana camara*. The flowers of this plant are found in many beautiful colors. The perfume of flowers and leaves is a sort of “*tutti-frutti-like*” smell that some people do not appreciate. However, the blooming is spectacular, with rich and extremely abundant flowers produced from late spring to fall, also in temperate areas. (**C**,**D**) Plants of marigold may belong to genus *Calendula* or *Tagetes*, and with respect to this which plant is used for which purpose is somewhat confusing. However, several species of *Tagetes erecta* are known, such as: African marigold, Aztec marigold, and French marigold, depending on their geographic origin. In any case, phyto-pharmacological studies have suggested that these plant extracts may have anti-viral, anti-genotoxic, and anti-inflammatory properties in vitro, and alcoholic extracts showed antibacterial activity and antifungal activities. In some areas of the world, *Calendula* petals and extracts are used as cosmetics, probably because they contain saponins, resins, and essential oils. In some areas of the world, they also find a culinary usage. (**E**) Chemical formula of the madecassol (molecular mass: 975.12) which is also known as madecassoside (official name) or asiticoside. It is a triterpenoid compound found in *Centella asiatica* and is used as a herbal medicament in traditional African medicine but also in Chinese and Ayurvedic medicine. Madecassoside has, allegedly, many biological activities, including anti-inflammatory, wound healing, and antioxidant activities. The molecule is claimed to have a neuroprotective action, and therefore may reduce stroke-derived damages. Madecassoside inhibits melanin synthesis, thus blocking ultraviolet-induced inflammation. (**F**) Commercial products of Madecassol such as skin regenerating cream, cosmetic oil, and anti-acne-preparations.

In Figure 3A,B inflorescences of *Lantana camara* are shown. See Figure 3 also for pictorials and details referring to madecassol.

### 2.3. Katrafay

**Figure 4:**
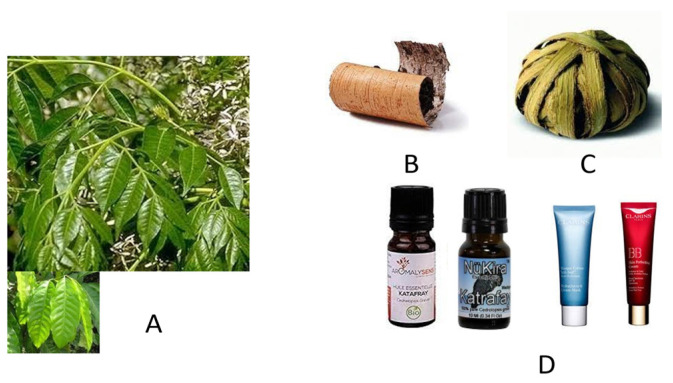
The plant and products of *Cedrelopsis grevei*. (**A**) The plant, with details of the leaves. (**B**) Rolled bark of the tree, often sold in this form. (**C**) Typical “boule de katrafay” available on local market and at Madagascan souvenir and handicraft shops. (**D**) Commercial preparations of essential oils and creams obtained from the bark of the tree. The essential oil is obtained by distillation of the bark of the plant. The wood is used to build the traditional houses, but also boats. The oil is used for massage, ambient deodorizing, and local body application. In any case, consumption as food is to be avoided. Katafray treatment is often associated with ylang ylang, another botanical fragrance common in Nosy Be, a large island on the Northern coast of Madagascar. However, ylang ylang, though commonly diffused in Madagascar, originates from Far East areas such as the Philippines, Myanmar and India.

Another popular herbal remedy of Madagascan origin is katrafay (sometimes spelled kathrafay, but also known under the name of katafa, as reported in the Histoire Physique, Naturelle et Politique de Madagascar 1893, http://legacy.tropicos.org/Publication/966?projectid=17, accessed on 23 June 2021) which is obtained from the plant *Cedrelopsis grevei* Brill (Ptaeroxylaceae). It was taxonomically classified as a Rutaceae (previously classified in the Meliaceae, then the Ptaeroxylaceae). This plant is an aromatic and endemic tree of Madagascar that can reach 15 m in height and one meter in diameter. However, it is also found in a bush-like form of smaller dimensions. The plant thrives in the dry areas of the West coast in the provinces of Tuléar, Mahajanga and Antsiranana (Diego Suarez). It grows in dry, sub-arid and sub-humid bioclimates, on the West coast in the provinces of Toliara (Tuléar), Mahajanga and Antsiranana (Diego Suarez). The plant range stretches from sea level to nine hundred meters of altitude. The wood is also used for construction. Bark and leaves are administered in traditional medicine, as they are or in various other forms; they are typically sold in local markets as a ball composed by intertwined twigs containing fragments of bark and leaves (they are also found in souvenir shops). One of the authors, (G.R.) used to purchase these manufacts at the Analakely (Zoma) market in Antananarivo. Prior to use, they should be soaked in boiling water and the resulting infusion used for wash or massage. An essential oil obtained from the plant is believed to relieve, among other afflictions, muscular fatigue. In any case, extracts of the trunk bark and leaves are empirically used in Madagascar commonly and widely in a number of diversified situations, ranging from persistent catarrh to hypertension. The tonic, fortifying, anti-inflammatory, febrifuge, and antalgic properties of these preparations are well known in the popular local medicine. Most likely, these properties derive from the presence of alkaloids, coumarins and chromones present in the plant preparations. Essential oils of this plant are mainly produced from the leaves or the bark and extracts of katrafay are commercially available in the form of oil or aqueous extracts available at retailers of wellness natural products. Figure 4 Illustrates some details of the *Cedrelopsis grevei* plant and its products.

**Figure 5:**
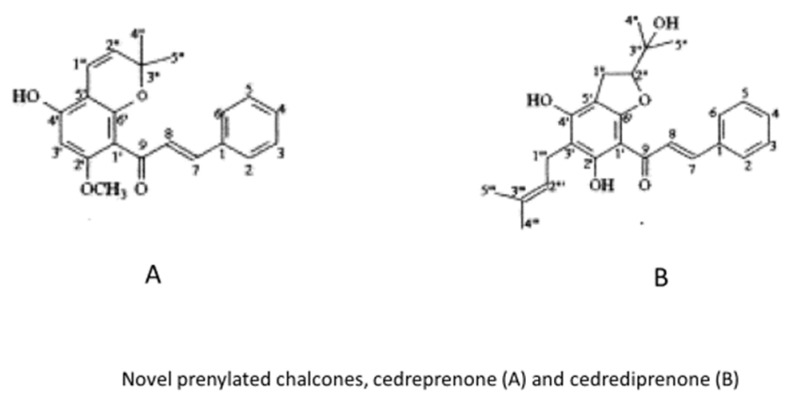
Specific prenylated chalcones isolated from *Cedrelopsis grevei*.

A biomolecular study of the effects of *C. grevei* extracts on age-related changes in systolic blood pressure and endothelial function was published. Results of this work implied that *C. grevei* prevents both increased blood pressure and age-related endothelial dysfunction, thus supporting the empirical use of these plant extracts to counteract mild hypertension which is often associated with aging. The composition of katrafay and the main physico-chemical features of its derivates show a very variable presence of presumably active principles; works on this specific subject were published [18,19,20]. However, two novel chalcones (Figure 5 for details) were isolated from the seeds of the plant [21].

Many natural compounds were obtained from the seeds of *C. grevei*. Some of them, such as uvangoletin, 5,7-dimethylpinocembrin, cardamonin, flavokawin B, 2′-methoxyhelikrausichalcone have been known and described for a long time. The novel prenylated chalcones, cedreprenone and cedrediprenone were presented in a relatively recent work. As also reported by the authors of the original work, this latter compound has been shown to exhibit superoxide scavenging properties [21].

## 3. Vanilla and Masonjoany

A work on the Madagascan officinal plants could not neglect to cite two more natural products originating from this country: vanilla and masonjoany.

In reality, neither product has a direct medicinal application. However, both are so diffused in Madagascar that their mention cannot be ignored. As a matter of fact, vanilla finds essentially a culinary usage, while masonjoany is used for cosmetic purposes. Therefore, a potential use in nanotechnological vehiculation to target recipient cells or individuals cannot be envisaged. With respect to potential therapeutic usages of vanilla orchid, it is worth mentioning that the sap from stems or beans of this plant, may cause skin dermatitis. As a matter of fact, this phenomenon has been frequently monitored in workers harvesting and exposing their bare skin to these vanilla plant products. Calcium oxalate crystals are believed to be the causative origin of contact dermatitis in vanilla plantation workers (https://pfaf.org/user/Plant.aspx?LatinName=Vanilla, accessed on 23 June 2021).

Masonjoany is the name of the powder obtained from the sap of the sandalwood of Madagascar. It is used in the Northern parts of the Red Island as a protective shield from solar irradiation. One of the authors (G.R.) received a personal communication from a woman interviewed in a street of Antananarivo, that this yellow paste is spread on the face of mourning widows, however this information must be considered unwarranted if not apocryphal.

### 3.1. Vanilla

Vanilla is obtained from orchids of the genus *Vanilla*, primarily obtained from pods of the Mexican species, flat-leaved vanilla. The plant actually originates from Central America, where pre-Columbian Mesoamericans cultivated the vine of the vanilla orchid. The Aztec word for vanilla is tlīlxochitl. Vanilla is a deformation of the Spanish word vaina (sheath); vanilla thus means little sheath. Three major species of vanilla are currently grown all over the world: the *V. planifolia* (also named *V. fragrans*), which thrives in Madagascar, La Réunion, and other tropical areas along the Indian Ocean; *V. tahitensis*, grown in the South Pacific; and *V. pompona*, found in the West Indies, Central America, and South America. However, the most appreciated product is the bourbon vanilla (after the former name of the island La Réunion, Île Bourbon) or Madagascar vanilla, which is produced in Madagascar and neighboring islands as well as in Indonesia. Madagascar and Indonesia provide two-thirds of the world’s supply of vanilla (*Vanilla planifolia*), but some African countries and Sri Lanka are becoming significant competing producers. Saffron is the only spice with a market value higher than vanilla, which owes its cost to the labor-expensive and cultivation/harvest procedures. Apart from aromatherapy, no medicinal applications for vanilla are known. Nevertheless, vanilla is highly appreciated in industrial and domestic baking, and perfume manufacture.

From a chemical point of view, vanilla essence is a complex mixture of several hundred different compounds, including vanillin, major acetaldehyde, acetic acid, furfural, hexanoic acid, 4-hydroxybenzaldehyde, eugenol, methyl cinnamate, and isobutyric acid. Synthetic essence consists of a solution of synthetic vanillin in ethanol. The chemical compound vanillin (4-hydroxy-3-methoxybenzaldehyde) is a contributor to the characteristic flavor and aroma of real vanilla and is the main flavor component of cured vanilla beans (https://cen.acs.org/articles/94/i36/problem-vanilla.html, accessed on 23 June 2021).

Vanillin was first isolated from vanilla pods by Gobley in 1858 [22]. Most artificial vanilla products contain vanillin, which can be produced synthetically from lignin, a natural polymer found in wood. Curiously, synthetic vanillin is also a by-product from the pulp used in papermaking, in which the lignin is broken down using sulfites or sulfates.

**Figure 6:**
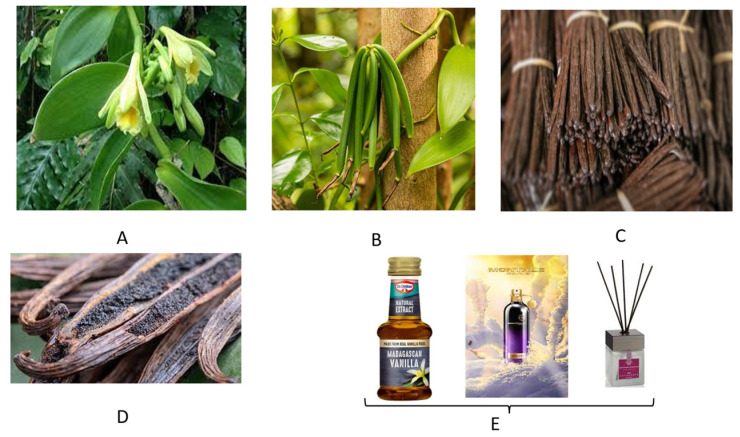
Details about the vanilla plant (*Vanilla planifolia*) and its products. (**A**,**B**) Details of the plant’s flower and pods. (**C**) Bundles of dried vanilla pods often offered for sale in the streets of many Madagascan sites. (**D**) Split vanilla pods showing their aromatic and valuable paste. (**E**) Commercially available vanilla products for cuisine (left), personal (center) and ambient deodorizing (right).

**Figure 7:**
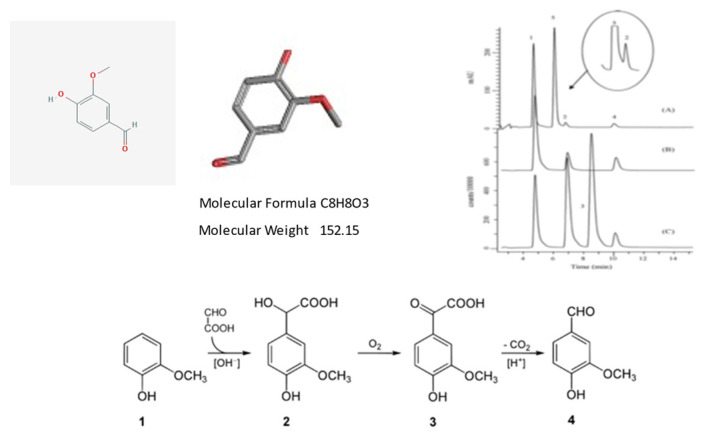
Some essential chemical features of the vanillin molecule.

However, vanillin is only one of 171 identified aromatic components of real vanilla fruits; Figure 6 and Figure 7 illustrate some essential feature of *Vanilla planifolia* and its products. In particular, this latter reports on some chemical/chromatographic features of the vanillin molecule.

Vanillin is the principal molecule contributing to the flavor and aroma of vanilla. Cured vanilla pods contain about 2% of dry weight vanillin. The molecule is also found in other plant species, such as *Leptotes bicolor*, an orchid species native to Paraguay and southern Brazil, and in the Southern Chinese red pine. Upper Panel, from left to right. Chemical structures and formulas of vanillin. Chromatographic analysis of vanillin and molecular analogues: vanillin (peak 1), ethyl-vanillin (peak 2) and coumarin (peak 3). Lower Panel. Vanillin is often produced semi-synthetically from eugenol as a starting molecule, or via complete chemical synthesis. The lower panel illustrates a process developed at Rhodia in the 1970s. This company specialized in chemistry, synthetic fibers and polymers, and has now merged into a large multinational concern (See https://en.wikipedia.org/wiki/Rhodia, accessed on 23 June 2021). In the original synthetic pathway, guaiacol (1) reacts with glyoxylic acid by electrophilic aromatic substitution. The resulting vanillylmandelic acid (2) is then converted by 4-Hydroxy-3-methoxyphenylglyoxylic acid (3) to vanillin (4) by oxidative decarboxylation. However, it should be pointed out that blind testing panels failed to distinguish between the flavors of synthetic vanillin from lignin and those from guaiacol, but can distinguish the odors of these two types of synthetic vanilla extracts. (For general details and chemical synthetic reactions the reader is pointed towards: https://en.wikipedia.org/wiki/Vanillin#:~:text=Vanillin%20is%20an%20organic%20compound,extract%20of%20the%20vanilla%20bean, accessed on 23 June 2021).

### 3.2. Masonjoany

**Figure 8:**
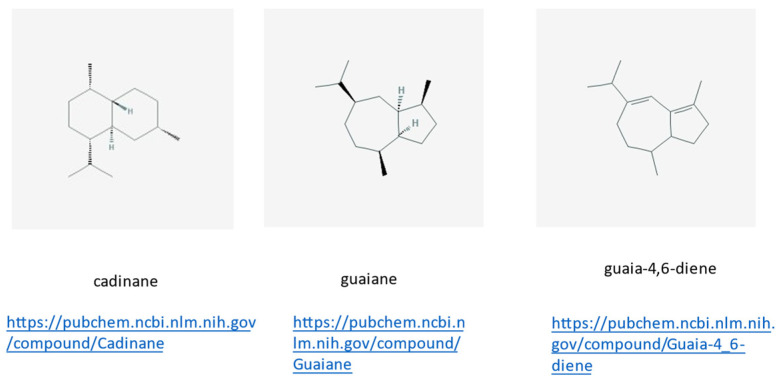
Three new cadinene- and guaiane-type sesquiterpenoids, all link accessed on 23 June 2021.

Masonjoany (pronounced in French as Massounjouany or Massunjun) is possibly one of the most baffling and elusive natural products used in Madagascar. Even the plant from which this product is obtained has an uncertain taxonomy, since the scientific name may be *Enterospermum madagascariensis,* but it is also classified with the botanical synonyms of *Santalina madagascariensis* or *Coptosperma madagascariensis*. It has different common names, such as Madagascar Santal, or Tabaky in the Southern areas of the country. The Masonjoany or sandalwood of Madagascar is a common endemic shrub that grows on the west coast of the island. One should be aware, however, that Madagascan sandalwood has nothing to share with sandalwood from India (*Santalum* species) utilized to produce essential oils and incense. In fact, stumps and roots contain a fragrant volatile oil which may remind one of a sandalwood-like fragrance. The tree *Santalina madagascariensis* grows in many different places, but in very localized areas of Madagascar. Scientific literature of the products of *E. madagascariensis* is scant, if existing. A work was published on the sesquiterpenoids content of this plant extract (Figure 8), but to our knowledge not much more is found in literature, as also stated by to the authors of the original work where these novel products obtained from *Enterospermum madagascariensis* were reported [23].

These compounds were isolated from the wood of *Enterospermum madagascariensis* (Rubiaceae) grown in Madagascar. Their structures were determined by well-established “classical” NMR techniques; for details see [23]. Below each chemical structure depiction, the www-site is reported for further information.

**Figure 9:**
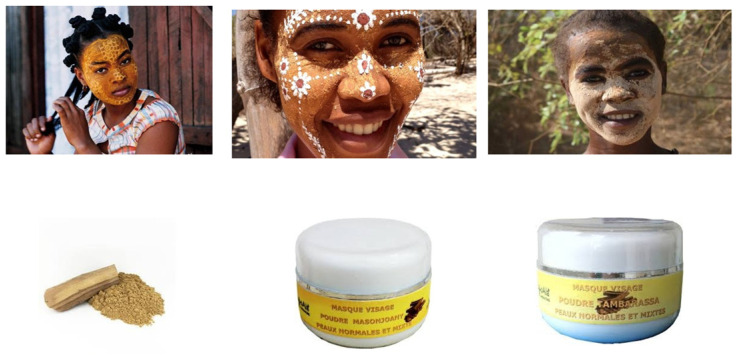
Face mask and commercially available Masonjoany products.

Traditionally, women of the Sakalava e Vezo people living on the coast of Nosy Be, Diego Suarez and Tuléar, fabricate this beauty and protective cream (which is also used in the Mascarene Islands). Traditionally, women grate a piece of Masonjoany on a coral stone with some water and oil. The paste obtained is applied to the face; the facial beauty mask is kept all day, but it crumbles away rather rapidly. This preparation is used as face painting, notably during the traditional ceremonies but also during the elections of Miss Madagascar (Figure 9). Several features are attributed to Masonjoany in addition to sunscreen, such as antiaging, elimination of skin impurities and generic skin cleansing properties. With respect to this, the reader should be aware that the private company Renala, based on the West coast of Madagascar, in the Morondava area, is a producer of virgin botanical natural ingredients and oils for cosmetic and nutraceutical industries, among which is Masonjoany.

Upper Panel, from left to right. Two Masonjoany tattoo-like facial decorations on young Malagasy women. The third example to the right is intended as a more traditional full mask protecting from solar irradiation. In this case the reader can observe how the creamy preparation becomes brittle as a consequence of rapid drying. Lower Panel, from left to right. Masonjoany powder obtained by grating the wood with a coral stone. Two jars of commercial sun protective screens.

In conclusion, the native Malagasy flora is rich, and the country presents a great biodiversity both in the plant and animal kingdom, as well as in ethnology; therefore, the amount of ethnobotanical information is extensive. To give a final example, various plants of the *Dianella* genus are utilized differently by the Tanala and Betsimisaraka people. In the Ranomafana region, Tanala people brew a tea from the leaves which is used to combat dysentery, while in Maroantsetra, the Betsimisarakas prepare a tea from the roots and stem used to reduce fever. Curiously, in Madagascar, certain plants are traditionally used for treating men, while others are used exclusively for women, for example to treat general fatigue. Therefore, medicinal plants are indicated with high specificity. It has been claimed that a deeper analysis of such gender-specific plant “prescriptions” could shed some light on our understanding of the different effects of various compounds on the male and female physiology. Finally, an exhaustive and detailed review of Madagascan plants and their products as well as applications has been published [18].

## 4. Nanomaterials in Advanced Biomedicine: Therapies of the Future?

To be concise, we illustrate only some species of different nanoparticles; however, it should be borne in mind that number, nature, and sophistication of molecular carriers for the delivery of exogenous material into a living cell are diversified, serve different purposes, and become ever more numerous. Here, we restrict the discussion to cationic liposomes, cat-anionic vesicles, and single-walled carbon nanotubes. This choice is due to the fact that these supramolecular particles represent a field of major expertise in our or in collaborating laboratories.

Drug absorption into an organ results from a complex behavior depending upon the chemical structure of the compound to be delivered. This concept was developed with great foresight by Hermann Müller, one the founders of modern genetics who prompted the discovery of nanometric needles capable to act directly on DNA to modify its features. An analogous concept was taken up by Paul Ehrlich, a precursor of molecular medicine, who was convinced that with magic bullets (“zauberkugeln”, according to the German definition) the same target could be reached. It follows that the features of a carrier are optimal when it behaves like a projectile hitting the target bull’s eye. This represents the major challenge and aim in designing the chemico-physical features of cargo supramolecular carriers. Which features should these supramolecular entities have to reach these aims? Cargos should guarantee the delivery (and uptake) of effective molecules which can have different structural and chemical characteristics. This aspect concerns, for instance, the optimal solubility of the drug to be delivered. Drugs are often transformed into acid salts when targeted to strongly polar tissues, or they may form complexes with crown ethers. Sophisticated methods rely on drug transport by gels and dendrimers carbon nanotubes, to mention but a few [24,25,26,27,28]. Carbon nanotubes and graphene composites are raising an enormous interest for their potential usage as cargo molecules both in cultured cells and in whole organisms, even though some concern exists about their environmental and health impacts [29,30].

### Liposomes, Cat-Anionic Vesicles, and Nanotubes

Biocompatibility, possibility of selective targeting and advantageous cost/production ratio play an important role in their selection as therapeutic agents. The validity of liposomes as cargo particles is mainly due to their flexibility, since they may function as vehicles for DNA, RNA, and proteins as well as small molecules such as hormones, natural compounds and/or drugs also of botanical origin. Liposomes based on cationic lipids are not found in nature but are synthesized in the chemistry laboratory: they possess a net positive charge and therefore may promptly interact with negatively charged cell membrane and nucleic acids [26,29,30,31,32]. The possibility of using liposomes in cancer therapy exists, since cationic liposomes are able to specifically deliver their payload to embryologically different tissues. Moreover, the interaction of this type of liposome does not interfere with the morpho-functional features of the target cells, as results from optical and scanning force imaging as well as NMR-metabolomic studies [31]. Finally, the antibacterial action of cationic liposomes has also been examined in antibiotic resistant pathogenic microorganisms. This action may derive from the ability of liposomes to increase the bacterial membrane permeability, with consequent higher susceptibility to drug uptake.

**Figure 10:**
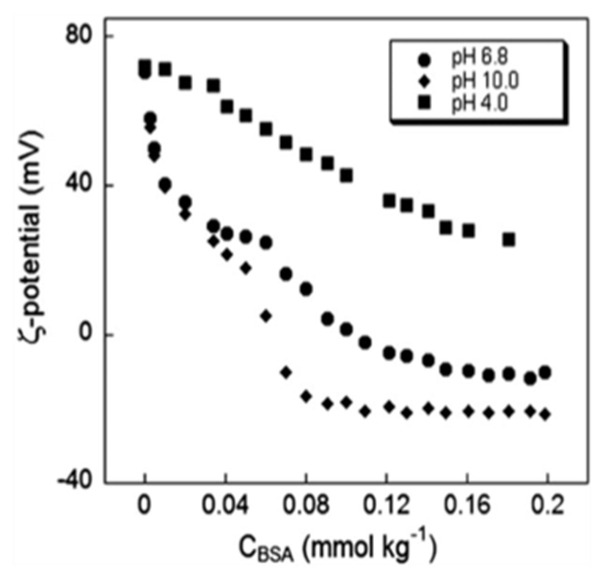
Essential chemico-physical parameters of a typical cat-anionic vesicle.

Catanionic vesicles are supramolecular aggregates formed by mixing in non-stoichiometric ratios of cat-ionic and anionic surfactant species [32]. Surfactants of opposite charge tend to aggregate in aqueous polar solvents (see the classical work by Israelachvili and collaborators for an extensive discussion [33]). As in the case of liposomes, vesicles interact with nucleic acids and other biopolymers. The nature of the complex vesicle/macromolecule, or lipoplex as it is also known, is of crucial importance in biotechnology and biomedicine, as they can deliver exogenous material across the cell membrane without causing a permanent or serious damage. However, vesicles may show a cytotoxic effect, which is directly related to time of exposure and dose of administration, as well as to the nature of the composing cat-anionic moieties which to some extent may determine the more or less pronounced cytotoxicity of the particle. In any case, cytotoxicity evaluations and biological viability has been evaluated from our and other authors: in particular, results from our laboratories show that the transfected cat-anionic/RNA lipoplex is efficiently translated into protein [34,35,36,37,38,39,40,41,42]. For a chemico-physical characterization of cat-anionic vesicles, see Figure 10 and reference [43].

There existed a surface charge density, expressed as zeta potential, of dispersions of cat-anionic vesicles to which bovine serum albumin was added. Experiments were performed at three pH values, and 25 °C.

Carbon nanotubes are graphene sheets organized in cylindrical structures, and they may be single- or multi-walled tube-like structures. Nanotubes have an astonishingly high potential for application in biochemistry, nanomedicine, pharmacology, and industry such as avionics as well as space engineering (see, for instance: [44,45,46,47,48]). However, we will limit the discussion to drug delivery and potential therapy. With respect to this, biocompatibility of carbon nanotubes, as well as other nanoparticles, must be evaluated in biological contexts to assess toxicity and immuno-tolerance, just to mention two fundamental aspects of the overall biocompatibility. The membrane is the first barrier encountered by the nanotube upon cell entry, therefore due to the scarce dispersibility of nanotubes in aqueous environments, they mainly establish van der Waals interactions that may eventually cause cell membrane damage [49,50]; however, non-covalent carbon nanotubes/BSA complexes did not alter the cell viability in murine fibroblasts, human embryonic kidney cells and murine macrophages. The supramolecular organization of nanotubes plays a role in their biocompatibility. For instance, nanotube crossing of the plasma membrane causes no alterations of the dielectric parameters of the cell membrane, which indicates that, at least at the first encounter, the nanoparticles did not damage the overall membrane structure, function, and permeability [34,35,36,37,51,52].

Graphene is at the basis of the nanotube technology: it consists of a one-atom-thick planar sheet of carbon atoms, which may be densely packed in a honeycomb crystal lattice. These structures are organized in different configurations derived from the basic structural carbon allotropes. Graphene-based nanotechnology represents nowadays an area of scientific research and industrial applications in full expansion, from material sciences to innovative and diverse applications such as electronics, photonics, composite materials, energy generation and storage and sensors as well as biological applications; for reviews see: [53,54,55,56]. A side problem, however, may be represented by the potential environmental impact of these new nanomaterials. Results are controversial, since the biological response often depends on the intrinsic structural nature of graphene. Data exist that these nanoparticles are essentially non-toxic in different animal models such as bacteria, lower crustaceans, nematodes, and amphibians. However, data from our laboratory show that the life span of these latter organisms did not seem to be affected, even though their growth rate was slower, possibly because of digestive and respiratory problems causing exchange gas dysfunctions [57,58,59,60,61,62,63,64,65,66,67,68,69].

## 5. Conclusive Remarks

A “classical” book describes fifty-eight medicinal plants found on the regular Friday (Zoma, in Malagasy) market on Analakely, Antananarivo [11]. Also, an extensive and detailed review describes analytically sixty-eight Madagascan plant species with medicinal use, even though the biomolecular mechanisms underlying their action is not taken into account [20]; this is no flaw of the paper, it is simply outside of the work’s aims. Regionally separated cultural groups often use and prepare plants following diverse “protocols”. Thirty-nine of them have been biochemically studied; thirty-four of the medicinal plants are not indigenous but were introduced to Madagascar. Many of them have proliferated in secondary forests or botanically disturbed habitats; this especially applies to the lantana, which has a highly invasive behavior. However, medicinal practice on the island is evolving and resorts to new species which are included into the Madagascan ethnopharmacological system.
